# 

**Published:** 1896-06

**Authors:** 


					﻿CONTENTS
ORIGINAL ARTICLES—
Dangers Which Threaten the Use of Cod Liver Oil. By Jno. T.
Winter, M. D., Washington, D. C.................... 285
Treatment of Abortion. By J. W. Long, M. D., Richmond, Va. 270
SELECTIONS AND ABSTRACTS—
Summer Complaint................................     273
A Case of Morphine and Opium Habit Cured............ 278
Leprosy in the United States and British Provinces.. 281
Report of Cases of Aneroticism in Women............. 282
Infection and Intoxication Through Food............. 284
Anti-Cholera Inoculation............................ 286
The Mechanical Treatment of Heart Disease........... 289
The Aachen Treatment of Syphilis.................... 292
SOCIETY REPORTS—
Richmond Academy of Medicine and Surgery..........  296
EDITORIAL—
The Atlanta Meeting of the American Medical Association.... 301
American Surgical Association..................     304
BOOK REVIEWS..........................................   306
SPECIAL NOTES.............................	............  308
Prescriptions  ...............................      316
				

